# Validation of the psychosocial consequences of screening in lung cancer questionnaire in the international lung screen trial Australian cohort

**DOI:** 10.1186/s12955-023-02225-8

**Published:** 2024-01-25

**Authors:** Asha Bonney, John Brodersen, Volkert Siersma, Katharine See, Henry M. Marshall, Daniel Steinfort, Louis Irving, Linda Lin, Jiashi Li, Siyuan Pang, Paul Fogarty, Fraser Brims, Annette McWilliams, Emily Stone, Stephen Lam, Kwun M. Fong, Renee Manser

**Affiliations:** 1 Department of Medicine, University of Melbourne, Melbourne, Australia; 2 Department of Respiratory and Sleep Medicine, Royal Melbourne Hospital, 300 Grattan Street, Parkville, VIC Australia; 3 Department of Public Health, Centre for General Practice, University of Copenhagen, Copenhagen, Denmark; 4 Primary Health Care Research Unit, Region Zealand, Copenhagen, Denmark; 5 Department of Social Medicine, The Research Unit for General Practice, University of Tromsø, Tromsø, Norway; 6 Respiratory Department, Northern Health, Melbourne, VIC Australia; 7 Department of Thoracic Medicine, University of Queensland Thoracic Research Centre, The Prince Charles Hospital, Chermside, QLD Australia; 8 Respiratory Department, Epworth Eastern Hospital, Box Hill, VIC Australia; 9 Department of Respiratory Medicine, Sir Charles Gairdner Hospital, Nedlands, WA Australia; 10 Department of Respiratory Medicine, Fiona Stanley Hospital, Murdoch, WA Australia; 11 University of Western Australia, Nedlands, Australia; 12 Department of Thoracic Medicine and Lung Transplantation, School of Clinical Medicine UNSW, St Vincent’s Hospital Sydney, Sydney, Australia; 13 Department of Medicine, The University of British Columbia, Vancouver, BC Canada

**Keywords:** Lung cancer, Screening, Computed tomography, Harms, Psychosocial impacts

## Abstract

**Background:**

Evaluation of psychosocial consequences of lung cancer screening with LDCT in high-risk populations has generally been performed using generic psychometric instruments. Such generic instruments have low coverage and low power to detect screening impacts. This study aims to validate an established lung cancer screening-specific questionnaire, Consequences Of Screening Lung Cancer (COS-LC), in Australian-English and describe early results from the baseline LDCT round of the International Lung Screen Trial (ILST).

**Methods:**

The Danish-version COS-LC was translated to Australian-English using the double panel method and field tested in Australian-ILST participants to examine content validity. A random sample of 200 participants were used to assess construct validity using Rasch item response theory models. Reliability was assessed using classical test theory. The COS-LC was administered to ILST participants at prespecified timepoints including at enrolment, dependent of screening results.

**Results:**

Minor linguistic alterations were made after initial translation of COS-LC to English. The COS-LC demonstrated good content validity and adequate construct validity using psychometric analysis. The four core scales fit the Rasch model, with only minor issues in five non-core scales which resolved with modification. 1129 Australian-ILST participants were included in the analysis, with minimal psychosocial impact observed shortly after baseline LDCT results.

**Conclusion:**

COS-LC is the first lung cancer screening-specific questionnaire to be validated in Australia and has demonstrated excellent psychometric properties. Early results did not demonstrate significant psychosocial impacts of screening. Longer-term follow-up is awaited and will be particularly pertinent given the announcement of an Australian National Lung Cancer Screening Program.

**Trial registration:**

NCT02871856.

**Supplementary Information:**

The online version contains supplementary material available at 10.1186/s12955-023-02225-8.

## Background

Lung cancer screening with low-dose computed tomography (LDCT) is currently recommended by several international associations [1, 2]. A meta-analysis of randomised controlled trials (RCTs) demonstrated a reduction in lung cancer-related mortality with LDCT compared to control groups in high-risk smoking populations [3]. However, whilst earlier diagnosis is associated with improved outcomes, screening also has unintended harms.

Of particular relevance to this paper is the limited characterisation of the potential for psychosocial consequences of lung cancer screening. There have been four lung cancer screening RCTs to date which have evaluated psychosocial consequences; the National Lung Screening Trial (NLST [4]), the Dutch-Belgian Nederlands-Leuvens Longkanker Screenings Onderzoek (NELSON [5]), the United Kingdom Lung Cancer Screening trial (UKLS [6]), and the Danish Lung Cancer Screening Trial (DLCST [7]). Only two studies (UKLS and DLCST) included the whole cohort in their psychosocial evaluation, and only one study (DLCST) used a condition-specific questionnaire. The DLCST reported more negative psychosocial consequences (affecting behaviour, dejection, and negative impact on sleep) in both the control and LDCT groups over 5years of annual screening using the condition-specific Consequences Of Screening Lung Cancer (COS-LC) questionnaire [8]. The DLCST trial performed a nested matched cohort study and observed that those who received false positive (FP) results had more negative psychosocial consequences compared with the control group and participants with true negatives in the short term [9]. FP results occur when a screen result is positive or indeterminate for cancer in a person who does not have cancer [10]. The rate of FPs from baseline LDCT in lung cancer screening in a meta-analysis of RCTs was 21% [3]. No significant long-term psychosocial consequences were noted in the DLCST, however the DLCST reported that those with FPs had an increased healthcare use in the years after their screening result [11, 12]. The UKLS administered to their whole cohort the Hospital Anxiety and Depression Scale (HADS), Revised 6-item Cancer Worry Scale (CWS-R), and Satisfaction with Decision Scale [13]. HADS scores were within the normal range for both groups, however, the control group reported lower Satisfaction with Decision to participate scores than the intervention group. Participants who were referred to multidisciplinary meetings in the screening arm experienced more short-term lung cancer distress, but no evidence of long-term consequences.

In the NLST, only 16 of the 23 sites invited participants in the LDCT screening arm to complete the State-Trait Anxiety Inventory (STAI) and Short Form 36-item questionnaire (SF-36) [14]. There was likely no difference between the groups (participants with true positive scans, scans with significant incidental findings, and negative LDCTs) in health-related quality of life (HRQoL) and anxiety measures [3]. The NELSON study included a random sample of 733 participants from each trial arm (LDCT screening and control) [15]. They used the Short Form 12-item questionnaire (SF-12), STAI, and Impact of Event Scale (IES). Participants with intermediate LDCT results had an elevated cancer-specific distress post result at two months, with no differences in measures between the groups at 2years.

Another study, Pan-Canadian Early Detection of Lung Cancer Study, assessed HRQoL using the SF-12 questionnaire, the EuroQol questionnaire, and the STAI [16]. LDCT screening was reported to have no overall impact on HRQoL, however a portion of participants were noted to have increased anxiety levels (number needed to harm = 7) which persisted at 12 months.

A potential limitation of almost all studies to date is the use of generic questionnaires which assess HRQoL without the context of the underlying condition [17]. These questionnaires have not had their psychometric performance evaluated in the target population and risk not capturing what is relevant [18]. Some studies have attempted to solve this issue by using multiple generic HRQoL questionnaires, however, this can fail to completely address the question and can introduce redundancy with repetition. The COnsensus-based Standards for the selection of health Measurement INstruments (COSMIN) risk of bias checklist was developed to evaluate the quality of Patient-Reported Outcome Measures (PROMs) in systematic reviews [19]. A systematic review assessing quality of PROMs in the evaluation of psychosocial consequences in colorectal cancer reported that 90% of PROMs lacked content validity according to the COSMIN checklist [20].

The aims of this study were to validate an Australian-English version of the COS-LC and to describe the short-term psychosocial impacts of lung cancer screening among an Australian high-risk cohort participating in the International Lung Screen Trial (ILST; NCT02871856).

## Methods

### Questionnaire translation and content validity assessment

The COS-LC questionnaire consists of four core themes (anxiety, behaviour, dejection, and negative impact on sleep) and twelve lung cancer screening-specific themes (focus on symptoms, stigmatisation, introvert, harms of smoking, self-blame, lung cancer, calm, social relations, existential values, impulsivity, empathy, and regretful still smoking). The questionnaire is divided into two parts; part 1 can be used at any timepoint (before, during, and after screening), whereas part 2 incorporates longer term consequences and is administered after the screening result/diagnosis. In part 1, higher scores indicate poorer outcomes, however part 2 measures the absolute change in either direction.

The Danish COS-LC was translated to Australian-English in Denmark with a three-member bilingual panel. A lay panel in Melbourne, Australia of five participants aged 55 to 80 years old assessed the initial translation’s functionally and ease of understanding as part of a double panel translation process [21]. Participants of the lay panel were volunteers who were invited to this process via flyers in a local Australian centre’s outpatient clinic. The lay panel was balanced for participant sex, with at least one participant in each decade aged 55 to 80 years old. Participants were selected based on availability to attend the group session, with health professionals excluded from the process. The group interview lasted approximately 2.5 h.

### Study design and participants

**Fig. 1:**
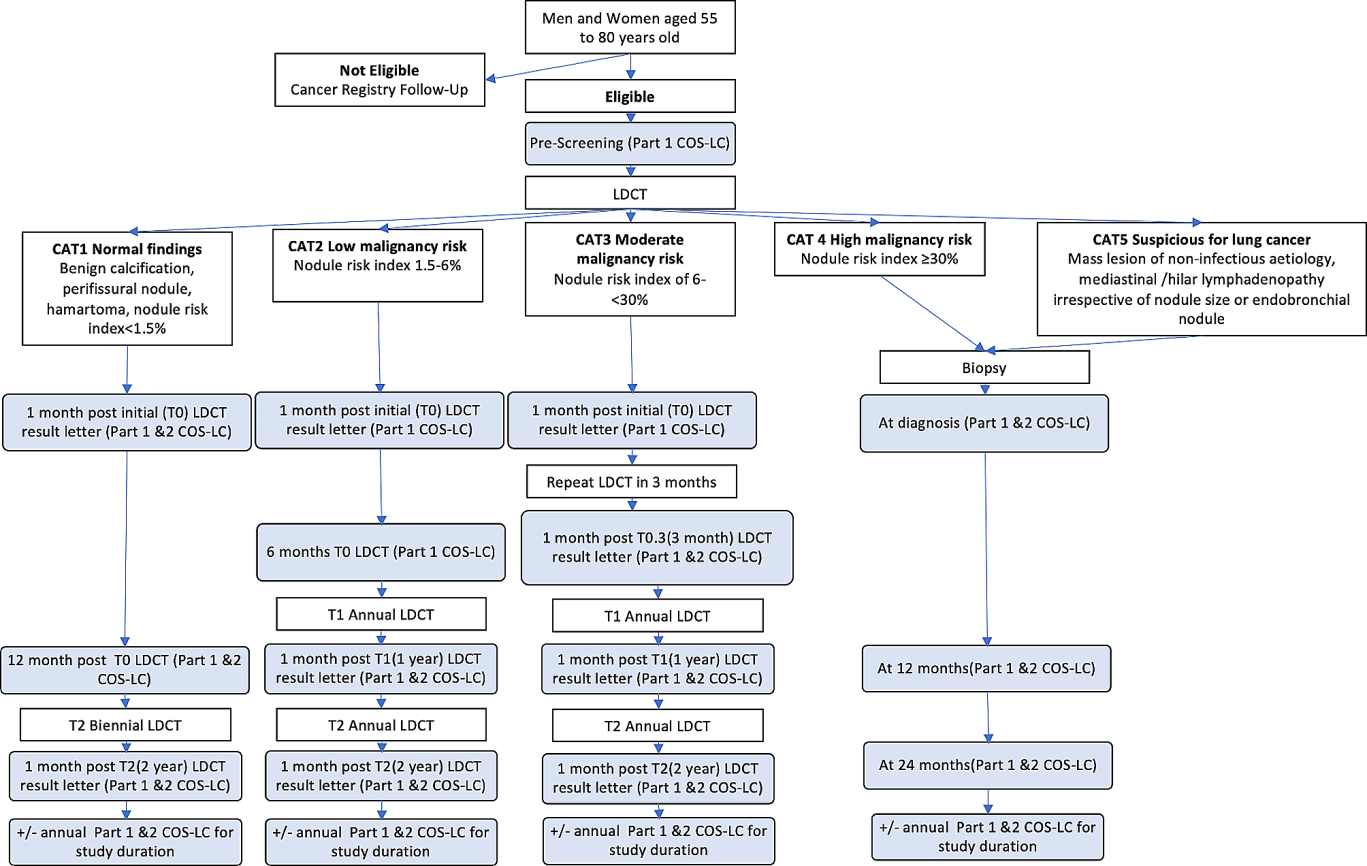
Initial LDCT (T0) ILST lung nodule management protocol with COS-LC timepoints

The ILST is a prospective cohort study with over 2000 Australian participants enrolled. Participants are men and women aged 55 to 80 years old who are current or former smokers with at least a PLCO_m2012_ 6-year risk of lung cancer ≥ 1.51% or ≥ 30 pack-year history of smoking. Participants undergo baseline LDCT and a 2-year LDCT. The full ILST protocol has been published [22]. LDCT results were given a category (CAT) based on likelihood of malignancy which then dictated the nodule management as per the protocol (Fig. 1 [22]) [23]. Definitions of CATs are also defined in Fig. 1.

Australian participants, across five centres in four states, were invited to participate in the quality-of-life study. The COS-LC questionnaires were administered as described in Fig. 1 via paper questionnaires or via electronic surveys. Each site collected their responses prospectively.

### Data analysis

For confounder adjustment, age, sex, smoking status, pack-years, education level, work and PLCO_m2012_ scores were collected. Baseline differences between risk CATs were assessed using a non-parametric Kruskal-Wallis Monte Carlo test for continuously valued data and a Pearson chi-squared Monte Carlo test for categorical variables.

COS-LC validation was performed using a random sample of 200 Australian ILST participants, in accordance with COSMIN Risk of Bias checklist for PROMs [19]. Construct validity was assessed in Rasch item response theory models. Aspects of construct validity tested were: unidimensionality, local response dependency and differential item functioning with respect to the above listed covariates. Reliability was assessed using classical test theory (Cronbach’s alpha). The Benjamini-Hochberg procedure was used to adjust for multiple testing.

The COS-LC scales’ scores from the baseline pre-screening assessment and the assessment 1-month after result letter/diagnosis of the Australian ILST cohort were analysed in linear regression models. In these models the mean differences between CATs were estimated, both unadjusted and adjusted for sex, age, smoking status and pack-years, education, work status and PLCO, parameterised such that estimates at 1-month follow-up were interpreted as mean differences beyond differences at baseline. Potential bias because of differential attrition between CATs was dealt with by weighting the available observations by the inverse of the probability of this observation being present; the latter was estimated from logistic regression models using the available covariates. Inference was corrected for both the repeated measurements and the weighting using the method of generalized estimating equations.

Analyses were performed with SAS v9.4, except for the analyses in the Rasch models, which was performed in DIGRAM.

This study was funded by the National Health and Medical Research Council of Australia. Funding sources had no role in study design.

## Results

### Field testing

Minor linguistic alterations were made to 22 of the part 1 items and 1 of the part 2 following field testing. All items were found to be relevant by the participants and content validity of the COS-LC was established via field testing amongst ILST screening participants. No new items or scales were added to the COS-LC Australian-English version. Table 1 summarises the themes and items of the COS-LC. The complete part 1 and 2 questionnaires are presented in the supplementary materials (Questionnaires 1 and 2).

**Table 1:** Themes and items of the COS-LC

**Part 1**		
**Themes**		**Content of items. The number relates to order of appearance in the COS-LC.**
Anxiety		2. I have been worried about my future
		3. I have felt scared
		12. I have been upset
		13. I have felt restless
		14. I have been nervous
		23. I have felt terrified
		28. I have felt shocked
Behavioural		4. I have been irritable
		5. I have been quieter than usual
		8. I have found it hard to concentrate
		10. My appetite has changed
		17. I have felt withdrawn
		20. I have had difficulty with my work or other commitments
		22. I have had difficulty doing everyday things around the house
Sense of dejection		1.I have been worried
		9. I have felt time passed slowly
		11. I have felt sad
		15. I have been uneasy
		18 I have felt unable to cope
		19. I have been depressed
Sleep		6. I have slept badly
		16. It has taken a long time to fall asleep
		21. I have woken up far too early in the morning
		24 I have been awake most of the night
Focus on (airway) symptoms		26. I have been aware more than usual of my weight
		27. I have been more aware than usual of being short of breath
		37. I have considered going to my doctor
		40. I have had more colds than usual
		42. I have been more tired than usual
		45. I have been more aware than usual of when I cough
		47. I have felt unwell
		49. I have been more aware than usual of coughing up phlegm
Stigmatisation		32. I have been criticised more than usual by other people for having smoked all these years.
		34. I have felt more than usual that others have pointed their finger at me for having smoked all these years
		43. I have felt stigmatised more than usual for having smoked all these years
		46. I have felt more than usual that others have blamed me for having smoked all these years
Introvert		29. In the back of my mind, I have been more afraid of having lung cancer than usual
		31. I have felt insecure
		33. I have felt sorry for myself
		39. I have felt my situation was hopeless
		41. I have had mood swings
		44. I have kept my thoughts to myself
Harms of smoking		25. I have thought more than usual that smoking is harmful
		30. I have regretted more than usual having smoked all these years
Self-blame		35. I have blamed myself more than usual for having smoked all these years
		36. I have felt guilty more than usual for having smoked all these years
		38. I have been disappointed with myself more than usual for having smoked all these years
		48. I have been angry with myself more than usual for having smoked all these years
		50. I have been annoyed with myself more than usual for having smoked all these years
Single items		7. I have kept busy to take my mind off things
		51. I have felt less interest in sex
		52. How many days of sick leave have you had during the last week (or how many days have you been unable to perform usual duties if unemployed)
**Part 2**		
**Themes**	**Content of items. The number relates to order of appearance in the COS-LC.**	
Lung cancer	3. After the examinations my anxiety about lung cancer is	
	13. After the examinations my belief that I do not have lung cancer is	
Relaxed/calm	4. After the examinations I feel (relaxed)	
	9. After the examinations I feel (calm)	
	17. After the examination I feel (relieved)	
Social relations	6. After the examinations my relationship with my family is	
	7. After the examinations my relationship with friends are	
	8. After the examinations my relationship with other people are	
Existential values	1. After the examination I have thought about the broader aspects of life	
	2. After the examinations my enjoyment of life is	
	5. After the examination my thoughts about the future are	
	10. After the examinations my sense of wellbeing is	
	11. After the examinations my awareness of life is	
	12. After the examinations I value life	
Impulsivity	14. After the examination my energy level	
	16. After the examinations I have lived my life to the full	
	19. After the examination I am (impulsive)	
	21. After the examinations, my desire to try new and unfamiliar things is	
	22. After the examinations, my desire to try risky things is	
	23. After the examinations I have done things that have exceeded my own boundaries:	
Empathy	15. After the examinations my sense of responsibility for my family is	
	18. After the examination I understand other people’s problems	
	20. After the examinations, my ability to listen to other people’s problems is	
Regretful still smoking (If yes to Q24. Do you smoke?)	25. After the examinations I have thought about quitting smoking	
	26. After the examinations I have felt guilty for smoking	
	27. After the examinations I have been irritated at myself for smoking	
	28. After the examinations I have been disappointed in myself for smoking.	
	29. After the examinations my view of myself as a smoker has changed	
	30. After the examinations I regret that I smoke	

### Baseline characteristics

A total of 1129 participants out of 1130 participants from three of the five (two Melbourne and one Brisbane) Australian ILST sites had data available at the time of analysis. One Melbourne participant did not complete the COS-LC questionnaires due to a language barrier. Only 11 participants (< 1%) identified as First Nations. The sample represented 54% (1129/2099) of Australian ILST participants. Centres were metropolitan hospitals, however some participants did attend from rural settings. The median cohort age was 63years old (IQR 59-69years old), with a lower median age in CAT1 compared to other groups. There was an overrepresentation of male participants (80%) in this study, with variability in distribution between categories. PLCO_m2012_score was lowest in participants with CAT1 LDCT results. There were no significant differences in covariates between groups otherwise, with demographics presented in Table 2.

**Table 2:** Demographics

	CT Result Lung Cancer Risk Category						
	**1.Normal findings**	**2. Low malignancy risk**	**3.Moderate malignancy risk**	**4. High malignancy risk**	**5. Suspicious for lung cancer**	**Total**	***p***-value*
**Number of participants**, n (%)	893 (79)	134 (12)	69 (6)	16 (1)	17 (2)	1129	
**Age (years)**, *Median [IQR]*	63, [58, 68]	65, [60,70]	67, [61, 72]	67, [62, 73]	68, [61, 72]	63, [59, 69]	0.0004
**Sex**, *n (%)*							0.0367
Female	153 (17)	36 (27)	24 (35)	4 (25)	6 (35)	223 (20)	
Male	740 (83)	98 (73)	45 (65)	12 (75)	11 (65)	906 (80)	
**Smoking Status**, *n (%)*							0.9439
Current	449 (50)	68 (51)	38 (55)	9 (56)	9 (53)	573 (51)	
Former	441 (49)	66 (49)	31 (45)	7 (44)	8 (47)	553 (49)	
Missing						3 (< 1)	
**Smoking (pack-years)**, *Median [IQR]*	44, [35, 56]	47, [35, 57]	49, [38, 63]	38, [30, 47]	45, [41, 60]	44, [35, 58]	0.0512
**Highest Attained Education**, *n (%)*							0.8385
8th grade or less	45 (5)	7 (5)	4 (6)	1 (6)	1 (6)	8 (5)	
9th to 11th grade	260 (29)	33 (25)	24 (35)	4 (25)	5 (29)	5326 (29)	
High school graduate	158 (18)	21 (16)	9 (13)	1 (6)	2 (12)	191 (17)	
Technical or Vocational Certificate	138 (15)	30 (22)	8 (12)	3 (19)	4 (24)	183 (16)	
Incomplete college/ university	101 (11)	11 (8)	10 (14)	3 (19)	3 (18)	128 (11)	
University graduate	116(13)	16 (12)	10 (14)	2 (13)	2 (12)	146 (13)	
Postgraduate	71(8)	16 (12)	4 (6)	2 (13)	0 (0)	93 (8)	
Missing						4 (< 1)	
**Work**, *n (%)*							0.2578
Working	335 (38)	33 (25)	14 (20)	4 (25)	4 (24)	390 (35)	
Retired	365 (41)	62 (46)	38 (55)	8 (50)	8 (47)	481 (43)	
Disabled	37 (4)	5 (4)	3 (4)	1 (6)	1 (6)	47 (4)	
Other	43 (5)	6 (4)	4 (6)	0 (0)	1 (6)	54 (5)	
Unemployed	26 (3)	9 (7)	2 (3)	0 (0)	0 (0)	37 (3)	
Extended Leave	4 (< 1)	0 (0)	0 (0)	0 (0)	0 (0)	4 (< 1)	
Missing						116 (10)	
**PLCO**_**m2012**_**score**, *Median [IQR]*	2.82, [1.84,4.58]	3.30, [2.01, 5.97]	4.71, [2.85, 6.87]	3.21, [2.46, 4.71]	3.38, [2.59, 8.47]	2.99, [1.91, 4,95]	< 0.0001

* *p*-value from a (non-parametric) Kruskal-Wallis Monte Carlo test (9999 resamplings) for continuous variables, or from a Pearson chi-squared Monte Carlo test (9999 resamplings) for categorical variables

### Psychometric analyses

Results of the psychometric analyses are presented in Table 3. The four core scales (anxiety, behaviour, dejection and sleep) all fit the Rasch model. There was some differential item functioning (DIF) with work status and negative impact on sleep, education level and focus on symptoms, smoking and harms of smoking, self-blame and empathy. The DIF disappeared when scales were modified to exclude the corresponding item.

**Table 3:** Conditional likelihood ratio (CLR) fit statistics and cronbach’s alpha for the 16 multi-item domains of the Consequences of screening-lung cancer (COS-LC) questionnaire

Scale (# of items)	CLR	df	***P*** value	Local Response Dependency	Differential item functioning	Cronbach’s alpha
**Consequences of Lung Cancer Screening Part 1**						
Anxiety (7)	34.29	18	0.0116*	2 & 3	Nil significant	0.824
Behaviour (7)	16.13	20	0.7085	4 &10, 4 & 20, 5 &17, 8 & 20	Nil significant	0.855
Dejection (6)	26.15	16	0.0520	Nil significant	Nil significant	0.873
Negative impact on sleep (4)	24.53	11	0.0107*	6 & 16, 6 & 24, 16 & 21	Item 21 & Work	0.849
*Negative impact on sleep (-21) (3)*	*11.72*	*8*	*0.1640*	*16 & 24*	*Nil significant*	*0.854*
Focus on symptoms (8)	62.97	22	< 0.0001*	26 & 37, 26 & 40, 26 & 45, 26 & 47, 42 & 47, 45 & 49	Item 40 & Education	0.739
*Focus on symptoms (-40) (7)*	*62.38*	*19*	< 0.0001*	*26 & 37, 26 & 45, 26 & 49, 42 & 47, 45 & 49*	*Nil significant*	*0.747*
Stigmatisation (4)	10.06	11	0.5253	Nil significant	Nil significant	0.873
Introvert (6)	26.30	17	0.0691	39 & 44	Nil significant	0.754
Harms of smoking (2)	12.34	5	0.0304	-	Item 25 & Smoking, Item 30 & Smoking	0.820
Self-Blame (5)	32.46	14	0.0034*	35 & 36, 35 & 38, 35 & 48, 35 & 50, 36 & 38, 36 & 48, 38 & 50, 48 & 50	Item 35 & Smoking	0.955
*Self-blame (-35) (4)*	*5.25*	*11*	*0.9185*	*36 & 38, 36 & 48, 48 & 50*	*Nil significant*	*0.951*
**Consequences of Lung Cancer Screening Part 2**						
Lung cancer (2)	1.84	3	0.6059	-	Nil significant	0.671
Relaxed/Calm (3)	6.28	5	0.2796	Nil significant	Nil significant	0.731
Social relations (3)	1.14	3	0.7685	Nil significant	Nil significant	0.817
Existential values (6)	15.65	11	0.1547	11 & 12	Nil significant	0.853
Impulsivity (6)	1.91	11	0.9988	Nil significant	Nil significant	0.778
Empathy (3)	11.18	4	0.0247	18 & 20	Item 15 & Smoking, Item 18 & Smoking	0.698
*Empathy (-15) (2)*	*0.00*	*2*	*0.9999*	*Nil significant*	*Nil significant*	*0.693*
*Empathy (-18) (2)*	*0.00*	*2*	*0.9994*	*Nil significant*	*Nil significant*	*0.540*
Regretful about still smoking (6)	24.18	5	0.0002*	27 & 28	Nil significant	0.832

*Benjamini-Hochberg rejects all *p*-values less than 0.0143 to control the false discovery rate at 0.05

### Baseline and 1-month responses

Mean scores (adjusted for age, sex, smoking status, pack-years, education, employment, PLCOm2012 score) of part 1 and part 2 are presented in Tables 4 and 5 respectively. Unadjusted mean sores are presented in supplementary materials (table S1 and S2). There was no significant difference in any of the themes (anxiety, behavioural, sense of dejection, sleep, focus on symptoms, stigmatisation, introvert, harms of smoking, self-blame, keeping busy, and interest in sex) using the unmodified scales across categories and time.

**Table 4:** Adjusted mean scores and mean difference in scores from baseline and after T0 (baseline CT) results for COS-LC Part 1

	Baseline*			Post CT results **		
Scale	Mean scores	95% CI	***p***-value (difference between CATs before screening)	Mean score change	95% CI	***p***-value (difference between CATs over interval)
1. Anxiety (0–21)			0.42			0.61
*CAT1*	0	0, 0		0.04	-0.14, 0.21	
*CAT2*	-0.10	-0.57, 0.37		0.29	-0.22, 0.80	
*CAT3*	0.17	-0.56, 2.06		0.45	-0.28, 1.17	
*CAT4*	0.73	-0.61, 2.06		0.50	-1.58, 2.58	
*CAT5*	-0.85	-1.91, 0.22		0.36	-0.26, 0.97	
2. Behavioural (0–21)			0.34			0.65
*CAT1*	0	0, 0		0.41	0.20, 0,62	
*CAT2*	-0.43	-0.89, 0.04		0.53	0.03, 1.02	
*CAT3*	0.15	-0.57, 0.87		0.67	-0.36, 1.71	
*CAT4*	0.47	-1.52, 2.46		1.22	-0.44, 2.87	
*CAT5*	-0.47	-1.90, 0.96		1.25	-0.15, 2.66	
3. Sense of dejection (0–18)			0.79			0.65
*CAT1*	0	0,0		0.16	-0.02, 0.34	
*CAT2*	-0.19	-0.65, 0.28		0.30	-0.19, 0.78	
*CAT3*	0.02	-0.66, 0.69		0.44	-0.27, 1.15	
*CAT4*	0.05	-1.10, 1.20		-0.39	-1.47, 0.70	
*CAT5*	-0.67	-1.84, 0.49		0.47	-0.10, 1.04	
4. Sleep (0–12)			0.53			0.10
*CAT1*	0	0, 0		-0.02	-0.19, 0.15	
*CAT2*	-0.20	-0.64, 0.25		0.29	-0.08, 0.66	
*CAT3*	0.48	-0.27, 1.23		0.07	-0.75, 0.89	
*CAT4*	-0.23	-1.21, 0.76		0.89	-0.08, 1.85	
*CAT5*	-1.28	-2.06, -0.51		1.52	0.28, 2.76	
4.1 Sleep (0–9) modified scale			0.51			0.04
*CAT1*	0	0, 0		-0.05	-0.19, 0.08	
*CAT2*	-0.26	-0.60, 0.09		0.33	0.04, 0.62	
*CAT3*	0.48	-0.16, 1.11		0.08	-0.63, 0.79	
*CAT4*	-0.31	-1.08, 0.46		0.50	-0.18, 1.19	
*CAT5*	-0.94	-1.54, -0.34		1.48	0.22, 2.73	
5. Focus on symptoms (0–24)			0.47			0.06
*CAT1*	0	0,0		0.16	-0.08, 0.39	
*CAT2*	-0.28	-0.96, 0.39		0.40	-0.25, 1.06	
*CAT3*	0.05	-0.91, 1.01		1.25	0.38, 2.13	
*CAT4*	-1.47	-2.72, -0.22		1.88	0.28, 3.49	
*CAT5*	-1.03	-2.79, 0.73		1.47	-0.96, 3.90	
5.1 Focus on symptoms (0–21) modified scale			0.64			0.05
*CAT1*	0	0, 0		0.11	-0.11, 0.33	
*CAT2*	-0.24	-0.89, 0.42		0.44	-0.20, 1.07	
*CAT3*	0.01	-0.92, 0.94		1.13	0.31, 1.96	
*CAT4*	-1.38	-2.64, -0.13		1.87	0.27, 3.48	
*CAT5*	-0.92	-2.65, 0.81		1.46	-0.95, 3.87	
6. Stigmatisation (0–12)			0.90			0.55
*CAT1*	0	0, 0		0.05	-0.08, 0.18	
*CAT2*	-0.01	-0.43, 0.41		-0.03	-0.44, 0.38	
*CAT3*	0.27	-0.34, 0.87		-0.03	-0.55, 0.50	
*CAT4*	-0.16	-0.76, 0.44		0.06	-1.16, 1.28	
*CAT5*	0.23	-0.46, 0.92		-0.40	-0.86, 0.06	
7. Introvert (0–18)			0.91			0.28
*CAT1*	0	0, 0		-0.04	-0.22, 0.14	
*CAT2*	-0.14	-0.71, 0.44		0.13	-0.40, 0.66	
*CAT3*	-0.10	-0.81, 0.60		0.77	-0.06, 1.60	
*CAT4*	-0.22	-1.46, 1.02		0.36	-0.60, 1.32	
*CAT5*	-0.82	-1.76, 0.12		0.88	-0.66, 2.41	
8. Harms of smoking (0–6)			0.82			0.50
*CAT1*	0	0, 0		-0.02	-0.13, 0.10	
*CAT2*	-0.10	-0.43, 0.24		0.09	-0.22, 0.39	
*CAT3*	0.10	-0.40, 0.59		0.34	-0.04, 0.72	
*CAT4*	-0.11	-0.99, 0.78		0.16	-0.55, 0.87	
*CAT5*	-0.12	-0.85, 0.62		0.39	-1.82, 2.60	
9. Self-blame (0–15)			0.57			0.13
*CAT1*	0	0,0		0.16	-0.08, 0.40	
*CAT2*	0.31	-0.47, 1.09		-0.00	-0.62, 0.61	
*CAT3*	-0.12	-1.12, 0.88		1.04	0.22, 1.86	
*CAT4*	-1.02	-1.99, -0.06		0.93	0.23, 1.62	
*CAT5*	-0.34	-1.58, 0.90		0.47	-1.29, 2.24	
9.1. Self-blame (0–12) *Modified scale*			0.60			0.19
*CAT1*	0	0, 0		0.12	-0.07, 0.31	
*CAT2*	0.26	-0.36, 0.88		0.02	-0.46, 0.50	
*CAT3*	-0.11	-0.92, 0.70		0.76	0.12, 1.39	
*CAT4*	-0.73	-1.57, 0.11		0.55	0.10, 0.99	
*CAT5*	-0.10	-1.12, 0.93		0.45	-1.10. 1.99	
Kept busy to take my mind off things (0–3)			0.65			0.65
*CAT1*	0	0, 0		0.06	-0.01, 0.12	
*CAT2*	-0.05	-0.19, 0.08		0.11	-0.04, 0.25	
*CAT3*	0.04	-0.15, 0.24		0.02	-0.27, 0.31	
*CAT4*	0.08	-0.37, 0.53		0.46	-0.19, 1.11	
*CAT5*	-0.07	-0.46, 0.31		-0.04	-0.23, 0.16	
Less interest in sex (0–3)			0.22			0.39
*CAT1*	0	0, 0		0.02	-0.06, 0.10	
*CAT2*	0.01	-0.22, 0.24		0.06	-0.15, 0.28	
*CAT3*	0.22	-0.14, 0.58		0.41	0.04, 0.78	
*CAT4*	-0.22	-0.54, 0.11		0.05	-0.33, 0.43	
*CAT5*	-0.08	-0.62, 0.45		0.12	-0.42, 0.66	

*Adjusted for age, sex, smoking status, pack-years, education, employment, PLCO_m2012_ score

**Adjusted for age, sex, smoking status, pack-years, education, employment, PLCO_m2012_ score and baseline responses

**Table 5:** Adjusted mean scores from after screening results for COS-LC Part 2

	Adjusted*		
Scales	Mean score	95% CI	***p***-value (difference between CATs after screening)
10. Lung cancer (0–4)			0.15
*CAT1*	0	0, 0	
*CAT2*	-0.49	-0.88, -0.10	
*CAT3*	0.69	-0.12, 1.50	
*CAT4*	0.08	-0.31, 0.47	
*CAT5*	0.26	-0.78, 1.29	
11. Relaxed/Calm (0–6)			0.06
*CAT1*	0	0, 0	
*CAT2*	-0.62	-1.07, -0.17	
*CAT3*	0.81	-0.01, 1.64	
*CAT4*	0.64	-0.61, 1.89	
*CAT5*	-0.26	-1.02, 0.49	
12. Social Relations (0–6)			0.05
*CAT1*	0	0, 0	
*CAT2*	0.05	-0.25, 0.35	
*CAT3*	0.72	0.30, 1.15	
*CAT4*	0.57	-0.31, 1.44	
*CAT5*	-0.05	-0.49, 0.39	
13. Existential values (0–12)			0.27
*CAT1*	0	0, 0	
*CAT2*	-0.29	-1.07, 0.49	
*CAT3*	0.88	-0.59, 2.35	
*CAT4*	1.87	0.02, 3.71	
*CAT5*	0.94	-0.33, 2.20	
14. Impulsivity (0–12)			0.31
*CAT1*	0	0, 0	
*CAT2*	0.01	-0.61, 0.64	
*CAT3*	1.15	0.12, 2.17	
*CAT4*	0.45	-0.36, 1.26	
*CAT5*	-0.38	-1.31, 0.56	
15. Empathy (0–6)			0.01
*CAT1*	0	0, 0	
*CAT2*	-0.04	-0.43, 0.35	
*CAT3*	0.93	0.41, 1.46	
*CAT4*	1.36	0.41, 2.31	
*CAT5*	0.78	0.28, 1.28	
15.1 Empathy (0–4) *Modified scale (-Q15)*			0.11
*CAT1*	0	0, 0	
*CAT2*	-0.02	-0.34, 0.30	
*CAT3*	0.55	0.08, 1.03	
*CAT4*	0.80	0.13, 1.48	
*CAT5*	0.39	-0.17, 0.95	
15.2 Empathy (0–4) *Modified scale (-Q18)*			0.02
*CAT1*	0	0, 0	
*CAT2*	-0.03	-0.28, 0.22	
*CAT3*	0.58	0.22, 0.93	
*CAT4*	0.85	0.24, 1.46	
*CAT5*	0.39	0.06, 0.71	
16. Regretful still smoking, if current smoker (0–5)			0.09
*CAT1*	0	0, 0	
*CAT2*	-1.67	-2.72, -0.62	
*CAT3*	-0.96	-2.41, 0.49	
*CAT4*			
*CAT5*	-2.06	-3.34, -0.78	

*Adjusted for age, sex, smoking status, pack-years, education, employment, PLCO_m2012_ score

There was a significant increase in scores on the modified sleep scale in the adjusted analysis for those in CAT2 and CAT5 following their T0 results. Part 2 did not demonstrate any significant difference between the categories across all themes except two. There was a significant difference in unadjusted analysis for the relaxed/calm theme, with CAT3 having the highest mean score, however there was no significant difference in the adjusted analysis. The adjusted analysis for empathy also demonstrated a significant difference between categories with CAT2 having a lower mean score and CAT3, 4 and 5 having higher scores compared to CAT1.

## Discussion

The Australian-English COS-LC questionnaire demonstrated high content and construct validity in an Australian lung cancer screening cohort. All core themes demonstrated excellent psychometric measurement properties, although four of the twelve lung cancer screening-specific themes demonstrated minor violations from the Rasch model. These violations were improved with modification of the items in each affected theme (sleep, focus on airway symptoms, self-blame, and empathy) as detailed in Table 3.

The early results from Australian-ILST demonstrate no major differences in HRQoL based on baseline CT results or over time from pre-screening to one month post initial LDCT results. Results of both the modified and unmodified scales were included for reference. Of note, in the DLCST participants with FP results were most adversely affected from a psychosocial perspective [9]. There have been studies demonstrating negative psychosocial impacts in other cancer screening programs with FP results. In breast cancer screening, more negative psychosocial consequences were noted in women who received FP results, with one study reporting persistent psychosocial consequences 12 to 14years after screening in women with FP mammograms [10, 24]. In colorectal cancer screening programs, one Danish study reported short-term and long-term psychosocial consequences of receiving a FP or diagnosis of polyps compared to a negative screening result using a condition-specific questionnaire [25]. There was no evidence of negative impacts from invitation to a colorectal cancer screening program [26].

The DLCST reported a significant increase in negative consequences in behaviour, dejection and sleep comparing round 1 with round 2 annual LDCT in the intervention and control group [8]. Although this increase was observed to decrease towards baseline in round 4 and 5 of annual LDCTs in behaviour and dejection scales. There was no similar trend in our Australian ILST cohort, though our reported follow up period was much shorter at approximately four weeks. There was a trend to poorer sleep in CAT5 participants after T0 results. It should be noted that there were differences between the Australian ILST cohort and DLCST baseline characteristics beyond country, with a higher portion of female, current smoker, and working participants in the DLCST [8]. The UKLS did report short-term results at 2 weeks of result of LDCT, although they did not use a condition-specific HRQoL measure, and as such the scope of psychosocial consequences assessed was limited [13]. They reported only an increase in anxiety in those referred to multidisciplinary meeting. When comparing to our CAT4 and 5 groups who would have had further investigation including possible multidisciplinary meeting discussion, our cohort did not have a significant increase in their anxiety scale, with confidence intervals that crossed 0.

Limitations of our early analysis of the Australian ILST cohort include higher CAT1 participant numbers compared to the other categories. The small numbers in CAT4 and 5 groups may have resulted in underestimation of differences. Additionally, the one-month timepoint was very early in the screening pathway of these participants, and consequently likely does not capture the total impact of the participant’s screening journey. There were also very few First Nations participants in our cohort which may limit extrapolation of the COS-LC to this population.

We specified this version as Australian-English as the translation of COS-LC was finalised in Australia. However, there were no significant colloquialisms incorporated and this version of the COS-LC is likely adaptable to other English-speaking settings.

Lung cancer screening is an evolving field with multiple ongoing studies evaluating implementation and efficacy in different populations. All systematic reviews to date which have evaluated psychosocial impacts of lung cancer screening have concluded that the available evidence is limited in the context of the number of studies, study design, and generic outcome measures [3, 27–29]. A key part of future research will be better characterising the psychosocial impact of screening on participants and designing consumer information and healthcare provider training that can ameliorate any potential negative consequences. Some psychosocial impacts of screening may in fact be positive and be beneficial to screening update and overall wellbeing. The COS-LC has been evaluated in two different international contexts and demonstrated high performance in measuring psychosocial consequences in both. While our version of the COS-LC was validated in Australia, it can be further tested and adapted to other settings. In future studies, condition-specific questionnaires, such as the COS-LC, should be used to enable adequate measurement of psychosocial impacts and more robust comparisons between different countries and participants to help inform the overall approach to screening.

## Conclusions

The COS-LC questionnaire has been validated in Australian-English in an Australian lung cancer screening cohort, demonstrating high content validity and adequate psychometric measurement properties. The early results from the Australian ILST cohort found minimal psychosocial impacts in the short-term using the COS-LC, a condition-specific questionnaire, however longer-term outcomes for the whole Australian ILST cohort are awaited.

### Electronic supplementary material

Below is the link to the electronic supplementary material.

Supplementary Material 1

## Data Availability

All data generated or analysed during this study are included in this published article and its supplementary information files. Requests for more information about data sharing should be directed to the corresponding author via email (asha.bonney@mh.org.au).

## Abbreviations

CAT: category
COS-LC: Consequences Of Screening Lung Cancer
COSMIN: COnsensus-based Standards for the selection of health Measurement INstruments
CWS-R: Revised 6-item Cancer Worry Scale
DIF: differential item functioning
DLCST: Danish Lung Cancer Screening Trial
FP: false positive
HADS: Hospital Anxiety and Depression Scale
HRQoL: health related quality of life
IES: Impact of Event Scale
ILST: International Lung Screen Trial
LDCT: low-dose computed tomography
NELSON: Nederlands-Leuvens Longkanker Screenings Onderzoek
NLST: National Lung Screening Trial
PROM: Patient-Reported Outcome Measures
RCT: randomised controlled trial
SF-12: Short Form 12-item questionnaire
SF-36: Short Form 36-item questionnaire
STAI: State-Trait Anxiety Inventory
UKLS: United Kingdom Lung cancer Screening
