# Supplementary material for: Validation of the psychosocial consequences of screening in lung cancer questionnaire in the international lung screen trial Australian cohort
Source: Health Qual Life Outcomes. 2024 Jan 25;22:10. doi: 10.1186/s12955-023-02225-8 (PMC10809555; doi:10.1186/s12955-023-02225-8)
Supplement: Supplementary file 1 — Supplementary Material 1 [file 12955_2023_2225_MOESM1_ESM.docx]

Questionnaire 1. Consequences Of Screening Lung Cancer Part 1

| To what extent – **over the last week** – have you experienced the following? | | | | | | | | | | | |
| --- | --- | --- | --- | --- | --- | --- | --- | --- | --- | --- | --- |
|  | | | | | Not at all | | A bit | | Quite a bit | | A lot |
| 1. I have been worried. | | | | | 🞏 | | 🞏 | | 🞏 | | 🞏 |
| 2. I have been worried about my future. | | | | | 🞏 | | 🞏 | | 🞏 | | 🞏 |
| 3. I have felt scared. | | | | | 🞏 | | 🞏 | | 🞏 | | 🞏 |
|  | | | | | Not at all | | A bit | | Quite a bit | | A lot |
| 4. I have been irritable. | | | | | 🞏 | | 🞏 | | 🞏 | | 🞏 |
| 5. I have been quieter than usual. | | | | | 🞏 | | 🞏 | | 🞏 | | 🞏 |
| 6. I have slept badly. | | | | | 🞏 | | 🞏 | | 🞏 | | 🞏 |
|  | | | | | Not at all | | A bit | | Quite a bit | | A lot |
| 7. I have kept busy to take my mind off things. | | | | | 🞏 | | 🞏 | | 🞏 | | 🞏 |
| 8. I have found it hard to concentrate. | | | | | 🞏 | | 🞏 | | 🞏 | | 🞏 |
| 9. I have felt time passed slowly. | | | | | 🞏 | | 🞏 | | 🞏 | | 🞏 |
| 10. My appetite has changed. | | | | | 🞏 | | 🞏 | | 🞏 | | 🞏 |
|  | | | | | Not at all | | A bit | | Quite a bit | | A lot |
| 11. I have felt sad. | | | | | 🞏 | | 🞏 | | 🞏 | | 🞏 |
| 12. I have been upset. | | | | | 🞏 | | 🞏 | | 🞏 | | 🞏 |
| 13. I have felt restless. | | | | | 🞏 | | 🞏 | | 🞏 | | 🞏 |
|  | | | | | Not at all | | A bit | | Quite a bit | | A lot |
| 14. I have been nervous. | | | | | 🞏 | | 🞏 | | 🞏 | | 🞏 |
| 15. I have been uneasy. | | | | | 🞏 | | 🞏 | | 🞏 | | 🞏 |
| 16. It has taken me a long time to fall asleep. | | | | | 🞏 | | 🞏 | | 🞏 | | 🞏 |
|  | | | | | Not at all | | A bit | | Quite a bit | | A lot |
| 17. I have felt withdrawn | | | | | 🞏 | | 🞏 | | 🞏 | | 🞏 |
| 18. I have felt unable to cope. | | | | | 🞏 | | 🞏 | | 🞏 | | 🞏 |
| 19. I have been depressed. | | | | | 🞏 | | 🞏 | | 🞏 | | 🞏 |
|  | | | | | Not at all | | A bit | | Quite a bit | | A lot |
| 20. I have had difficulty dealing with my work or other commitments. | | | | | 🞏 | | 🞏 | | 🞏 | | 🞏 |
| 21. I have woken up far too early in the morning. | | | | | 🞏 | | 🞏 | | 🞏 | | 🞏 |
| 22. I have had difficulty doing everyday things around the house. | | | | | 🞏 | | 🞏 | | 🞏 | | 🞏 |
| 23. I have felt terrified. | | | | | 🞏 | | 🞏 | | 🞏 | | 🞏 |
| 24. I have been awake most of the night. | | | | | 🞏 | | 🞏 | | 🞏 | | 🞏 |
|  | | | | | Not at all | | A bit | | Quite a bit | | A lot |
| 25. I have thought more than usual that smoking is harmful. | | | | | 🞏 | | 🞏 | | 🞏 | | 🞏 |
| 26. I have been aware more than usual of my weight. | | | | | 🞏 | | 🞏 | | 🞏 | | 🞏 |
| 27. I have been aware more than usual of being short of breath. | | | | | 🞏 | | 🞏 | | 🞏 | | 🞏 |
| 28. I have felt shocked. | | | | | 🞏 | | 🞏 | | 🞏 | | 🞏 |
|  | | | | | Not at all | | A bit | | Quite a bit | | A lot |
| 29. In the back of my mind, I have been more afraid of having lung cancer than usual. | | | | | 🞏 | | 🞏 | | 🞏 | | 🞏 |
| 30. I have regretted more than usual having smoked for all these years. | | | | | 🞏 | | 🞏 | | 🞏 | | 🞏 |
| 31. I have felt insecure. | | | | | 🞏 | | 🞏 | | 🞏 | | 🞏 |
|  | | | | |  | |  | |  | |  |
| To what extent – **over the last week** – have you experienced the following? | | | | | | | | | | | |
|  | | | | | Not at all | | A bit | | Quite a bit | | A lot |
| 32. I have been criticised more than usual by other people for having smoked all these years. | | | | | 🞏 | | 🞏 | | 🞏 | | 🞏 |
| 33. I have felt sorry for myself. | | | | | 🞏 | | 🞏 | | 🞏 | | 🞏 |
| 34. I have felt more than usual that others have pointed their finger at me for having smoked all these years. | | | | | 🞏 | | 🞏 | | 🞏 | | 🞏 |
| 35. I have blamed myself more than usual for having smoked all these years. | | | | | 🞏 | | 🞏 | | 🞏 | | 🞏 |
| 36. I have felt guilty more than usual for having smoked all these years. | | | | | 🞏 | | 🞏 | | 🞏 | | 🞏 |
|  | | | | | Not at all | | A bit | | Quite a bit | | A lot |
| 37. I have considered going to my doctor. | | | | | 🞏 | | 🞏 | | 🞏 | | 🞏 |
| 38. I have been disappointed with myself more than usual for having smoked all these years. | | | | | 🞏 | | 🞏 | | 🞏 | | 🞏 |
| 39. I have felt my situation was hopeless. | | | | | 🞏 | | 🞏 | | 🞏 | | 🞏 |
|  | | | | | Not at all | | A bit | | Quite a bit | | A lot |
| 40. I have had more colds than usual. | | | | | 🞏 | | 🞏 | | 🞏 | | 🞏 |
| 41. I have had mood swings. | | | | | 🞏 | | 🞏 | | 🞏 | | 🞏 |
| 42. I have been more tired than usual. | | | | | 🞏 | | 🞏 | | 🞏 | | 🞏 |
| 43. I have felt stigmatized more than usual for having smoked all these years. | | | | | 🞏 | | 🞏 | | 🞏 | | 🞏 |
| 44. I have kept my thoughts to myself. | | | | | 🞏 | | 🞏 | | 🞏 | | 🞏 |
|  | | | | | Not at all | | A bit | | Quite a bit | | A lot |
| 45. I have been more aware than usual of when I cough. | | | | | 🞏 | | 🞏 | | 🞏 | | 🞏 |
| 46. I have felt more than usual that others have blamed me for having smoked all these years. | | | | | 🞏 | | 🞏 | | 🞏 | | 🞏 |
| 47. I have felt unwell. | | | | | 🞏 | | 🞏 | | 🞏 | | 🞏 |
| 48. I have been angry with myself more than usual for having smoked all these years. | | | | | 🞏 | | 🞏 | | 🞏 | | 🞏 |
| 49. I have been more aware than usual of coughing up phlegm. | | | | | 🞏 | | 🞏 | | 🞏 | | 🞏 |
| 50. I have been annoyed with myself more than usual for having smoked all these years. | | | | | 🞏 | | 🞏 | | 🞏 | | 🞏 |
|  | | Not at all | | A bit | | Quite a bit | | A lot | | Not applicable | |
| 51. I have felt less interest in sex. | | 🞏 | | 🞏 | | 🞏 | | 🞏 | | 🞏 | |
| 52. How many days of sick leave have you had **during the last week**? (If you are not currently employed in paid work include days you were unable to perform your usual household/carer/volunteer duties) | | | | | | | | | | | |
| 0 days | 1 - 2 days | | 3 - 4 days | | | | | 5 or more days | | | |
| 🞏 | 🞏 | | 🞏 | | | | | 🞏 | | | |

Questionnaire 2. Consequences Of Screening Lung Cancer Part 2

| Taking everything into account, has your experience of the Lung Screening Programme caused any of the following:  **Please tick only one box for each question** | | | | |
| --- | --- | --- | --- | --- |
| 1. After the examinations I have thought about the broader aspects of life: | | | | |
| 🞏 | 🞏 | 🞏 | 🞏 | 🞏 |
| Much less | Less | **The same as before** | More | Much more |
| **Fewer thoughts about life** | |  | **More thoughts about life** | |
| 2. After the examinations my enjoyment of life is: | | | | |
| 🞏 | 🞏 | 🞏 | 🞏 | 🞏 |
| Much less | Less | **The same as before** | More | Much more |
| **Less enjoyment** | |  | **More enjoyment** | |
| 3. After the examinations my anxiety about lung cancer is: | | | | |
| 🞏 | 🞏 | 🞏 | 🞏 | 🞏 |
| Much greater | Greater | **The same as before** | Less | Much less |
| **Greater anxiety** | |  | **Less anxiety** | |
| 4. After the examinations I feel: | | | | |
| 🞏 | 🞏 | 🞏 | 🞏 | 🞏 |
| Much less relaxed | Less relaxed | **As relaxed as before** | More relaxed | Much more relaxed |
| **Less relaxed** | |  | **More relaxed** | |
| 5. After the examinations my thoughts about the future are: | | | | |
| 🞏 | 🞏 | 🞏 | 🞏 | 🞏 |
| Much more pessimistic | More pessimistic | **The same as before** | More optimistic | Much more optimistic |
| **More pessimistic** | |  | **More optimistic** | |

| Taking everything into account, has your experience of the Lung Screening Programme caused any of the following:  **Please tick only one box for each question** | | | | | |
| --- | --- | --- | --- | --- | --- |
| 1. After the examinations my relationship with my family is: | | | | | |
| 🞏 | 🞏 | | 🞏 | 🞏 | 🞏 |
| Much less close | Less close | | **The same as before** | Closer | Much closer |
| **Less close** | | |  | **Closer** | |
| 1. After the examinations my relationship with friends is: | | | | | |
| 🞏 | 🞏 | | 🞏 | 🞏 | 🞏 |
| Much less close | Less close | | **The same as before** | Closer | Much closer |
| **Less close** | | |  | **Closer** | |
| 1. After the examinations my relationship with other people is: | | | | | |
| 🞏 | | 🞏 | 🞏 | 🞏 | 🞏 |
| Much worse | | Worse | **The same as before** | Better | Much better |
| **Worse** | | |  | **Better** | |
| 1. After the examinations I feel: | | | | | |
| 🞏 | | 🞏 | 🞏 | 🞏 | 🞏 |
| Much less calm | | Less Calm | **As calm as before** | Calmer | Much calmer |
| **Less calm** | | |  | **Calmer** | |
| 1. After the examinations my sense of well-being is: | | | | | |
| 🞏 | | 🞏 | 🞏 | 🞏 | 🞏 |
| Much less | | Less | **The same as before** | Greater | Much greater |
| **Less sense of well-being** | | |  | **Greater sense of well-being** | |

| Taking everything into account, has your experience of the Lung Screening Programme caused any of the following:  **Please tick only one box for each question** | | | | | |
| --- | --- | --- | --- | --- | --- |
| 1. After the examinations my awareness of life is: | | | | | |
| 🞏 | 🞏 | 🞏 | 🞏 | | 🞏 |
| Much less | Less | **The same as before** | Greater | | Much greater |
| **Less awareness of life** | |  | **Greater awareness of life** | | |
| 1. After the examinations I value life: | | | | | |
| 🞏 | 🞏 | 🞏 | 🞏 | | 🞏 |
| Much less | Less | **The same as before** | More | | Much more |
| **Value life less** | |  | **Value life more** | | |
| 1. After the examinations my belief that I do **not** have lung cancer is: | | | | | |
| 🞏 | 🞏 | 🞏 | 🞏 | | 🞏 |
| Much less | Less | **The same as before** | Greater | | Much greater |
| **Less belief** | |  | **Greater belief** | | |
| 14. After the examinations my energy level is: | | | | | |
| 🞏 | 🞏 | 🞏 | 🞏 | 🞏 | |
| Much less | Less | **The same as before** | Greater | Much greater | |
| **Less energy** | |  | **More energy** | | |
| 15. After the examinations my sense of responsibility for my family is: | | | | | |
| 🞏 | 🞏 | 🞏 | 🞏 | 🞏 | |
| Much less | Less | **The same as before** | More | Much more | |
| **Less responsibility** | |  | **More responsibility** | | |

| Taking everything into account, has your experience of the Lung Screening Programme caused any of the following:  **Please tick only one box for each question** | | | | |
| --- | --- | --- | --- | --- |
| 16. After the examinations I have lived my life to the full: | | | | |
| 🞏 | 🞏 | 🞏 | 🞏 | 🞏 |
| Much less | Less | **The same as before** | More | Much more |
| **Lived life less** | |  | **Lived life more** | |
| 17. After the examinations I feel: | | | | |
| 🞏 | 🞏 | 🞏 | 🞏 | 🞏 |
| Much less  relieved | Less relieved | **As relieved**  **as before** | More relieved | Much more relieved |
| **Less relieved** | |  | **More relieved** | |
| 18. After the examinations I understand other people’s problems: | | | | |
| 🞏 | 🞏 | 🞏 | 🞏 | 🞏 |
| Much less | Less | **The same as before** | Better | Much better |
| **Less understanding** | |  | **Better understanding** | |
| 19. After the examinations I am: | | | | |
| 🞏 | 🞏 | 🞏 | 🞏 | 🞏 |
| Much less  impulsive | Less impulsive | **The same as before** | More impulsive | Much more  impulsive |
| **Less impulsive** | |  | **More impulsive** | |
| 20. After the examinations my ability to listen to other people’s problems is: | | | | |
| 🞏 | 🞏 | 🞏 | 🞏 | 🞏 |
| Much less | Less | **The same as before** | Greater | Much greater |
| **Less ability to listen** | |  | **Greater ability to listen** | |

| Taking everything into account, has your experience of the Lung Screening Programme caused any of the following:  **Please tick only one box for each question** | | | | |
| --- | --- | --- | --- | --- |
| 21. After the examinations my desire to try new and unfamiliar things is: | | | | |
| 🞏 | 🞏 | 🞏 | 🞏 | 🞏 |
| Much less | Less | **The same as before** | Greater | Much greater |
| **Less desire** | |  | **Greater desire** | |
| 22. After the examinations my desire to try risky things is: | | | | |
| 🞏 | 🞏 | 🞏 | 🞏 | 🞏 |
| Much less | Less | **The same as before** | Greater | Much greater |
| **Less desire** | |  | **Greater desire** | |
| 23. After the examinations I have done things that have exceeded my own boundaries: | | | | |
| 🞏 | 🞏 | 🞏 | 🞏 | 🞏 |
| Much less | Less | **The same as before** | More | Much more |
| **Less** | |  | **More** | |

| 24. Do you smoke? | Yes 🞏 | No 🞏 | | |
| --- | --- | --- | --- | --- |
| **If you smoke please complete the final items below** | | | | |
| Taking everything into account **after the examinations** X months ago, have you experienced the following :  **Please tick only one box for each question** | | | | |
|  | | | Yes | No |
| 25. After the examinations I have thought about quitting smoking. | | | 🞏 | 🞏 |
| 26. After the examinations I have felt guilty for smoking. | | | 🞏 | 🞏 |
| 27. After the examinations I have been irritated at myself for smoking. | | | 🞏 | 🞏 |
| 28. After the examinations I have been disappointed in myself for smoking. | | | 🞏 | 🞏 |
| 29. After the examinations my view of myself as a smoker has changed. | | | 🞏 | 🞏 |
| 30. After the examinations I regret that I smoke. | | | 🞏 | 🞏 |

Table S1. Unadjusted mean scores and mean difference in scores from baseline and after T0 (baseline CT) results for COS LC Part 1

|  | Baseline | | | | Post CT results | | | |
| --- | --- | --- | --- | --- | --- | --- | --- | --- |
| Scale | N | Mean scores | 95% CI | p-value (difference between CATs before screening) | N | Mean score change | 95% CI | p-value (difference between CATs over the screening interval) |
| 1. Anxiety (0-21) |  |  |  | 0.57 |  |  |  | 0.63 |
| *CAT1* | 854 | 2.00 | 1.82, 2.18 |  | 606 | 0.07 | -0.12, 0.27 |  |
| *CAT2* | 127 | 2.08 | 1.56, 2.59 |  | 97 | 0.15 | -0.36, 0.66 |  |
| *CAT3* | 67 | 2.10 | 1.40, 2.81 |  | 48 | 0.73 | -0.06, 1.52 |  |
| *CAT4* | 15 | 2.26 | 1.05, 3.48 |  | 7 | 0.49 | -1.15, 2.13 |  |
| *CAT5* | 15 | 1.46 | 0.33, 2.58 |  | 6 | 0.08 | -0.55, 0.72 |  |
| 2. Behavioural (0-21) |  |  |  | 0.56 |  |  |  | 0.51 |
| *CAT1* | 859 | 1.92 | 1.71, 2.12 |  | 607 | 0.45 | 0.23, 0.67 |  |
| *CAT2* | 131 | 1.67 | 1.20, 2.13 |  | 97 | 0.47 | -0.01, 0.96 |  |
| *CAT3* | 68 | 2.18 | 1.49, 2.87 |  | 45 | 1.03 | -0.01, 2.06 |  |
| *CAT4* | 16 | 2.01 | 0.34, 3.68 |  | 7 | 1.33 | -0.07, 2.72 |  |
| *CAT5* | 15 | 1.41 | 0.26, 2.55 |  | 6 | 1.11 | -0.21, 2.43 |  |
| 3. Sense of dejection (0-18) |  |  |  | 0.26 |  |  |  | 0.19 |
| *CAT1* | 865 | 1.93 | 1.74, 2.12 |  | 618 | 0.22 | 0.03, 0.41 |  |
| *CAT2* | 130 | 2.03 | 1.53, 2.54 |  | 96 | 0.12 | -0.35, 0.60 |  |
| *CAT3* | 68 | 2.05 | 1.41, 2.70 |  | 47 | 0.90 | 0.17, 1.63 |  |
| *CAT4* | 16 | 1.65 | 0.71, 2.60 |  | 7 | -0.51 | -1.24, 0.22 |  |
| *CAT5* | 15 | 1.37 | 0.42, 2.32 |  | 6 | 0.35 | -0.13, 0.84 |  |
| 4. Sleep (0-12) |  |  |  | 0.61 |  |  |  | 0.14 |
| *CAT1* | 874 | 2.06 | 1.89, 2.23 |  | 614 | 0.01 | -0.15, 0.18 |  |
| *CAT2* | 132 | 2.01 | 1.60, 2.43 |  | 96 | 0.29 | -0.08, 0.67 |  |
| *CAT3* | 68 | 2.56 | 1.89, 3.24 |  | 48 | 0.16 | -0.57, 0.88 |  |
| *CAT4* | 14 | 1.44 | 0.65, 2.23 |  | 8 | 1.02 | 0.11, 1.93 |  |
| *CAT5* | 16 | 1.28 | 0.57, 1.99 |  | 6 | 1.38 | -0.05, 2.80 |  |
| 4.1 Sleep (0-9) modified scale |  |  |  | 0.37 |  |  |  | 0.06 |
| *CAT1* | 875 | 1.48 | 1.35, 1.61 |  | 615 | -0.02 | -0.16, 0.11 |  |
| *CAT2* | 132 | 1.37 | 1.04, 1.70 |  | 97 | 0.33 | 0.03, 0.62 |  |
| *CAT3* | 68 | 1.99 | 1.40, 2.58 |  | 48 | 0.13 | -0.51, 0.77 |  |
| *CAT4* | 14 | 0.95 | 0.39, 1.51 |  | 8 | 0.59 | -0.04, 1.22 |  |
| *CAT5* | 16 | 0.94 | 0.43, 1.44 |  | 6 | 1.43 | -0.02, 2.89 |  |
| 5. Focus on symptoms (0-24) |  |  |  | 0.38 |  |  |  | 0.11 |
| *CAT1* | 859 | 3.59 | 3.34, 3.84 |  | 620 | 0.16 | -0.07, 0.39 |  |
| *CAT2* | 132 | 3.63 | 3.00, 4.26 |  | 95 | 0.36 | -0.27, 0.99 |  |
| *CAT3* | 66 | 3.83 | 2.91, 4.75 |  | 44 | 1.39 | 0.58, 2.19 |  |
| *CAT4* | 15 | 2.96 | 1.24, 4.67 |  | 8 | 0.28 | -2.22, 2.78 |  |
| *CAT5* | 15 | 3.89 | 1.90, 5.87 |  | 6 | 0.51 | -1.71, 2.73 |  |
| 5.1 Focus on symptoms (0-21) modified scale |  |  |  | 0.48 |  |  |  | 0.11 |
| *CAT1* | 860 | 3.50 | 3.26, 3.74 |  | 620 | 0.12 | -0.10, 0.34 |  |
| *CAT2* | 132 | 3.56 | 2.94, 4.18 |  | 95 | 0.37 | -0.24, 0.97 |  |
| *CAT3* | 66 | 3.67 | 2.78, 4.57 |  | 44 | 1.22 | 0.48, 1.97 |  |
| *CAT4* | 15 | 2.90 | 1.24, 4.57 |  | 8 | 0.38 | -1.98, 2.75 |  |
| *CAT5* | 15 | 3.83 | 1.86, 5.80 |  | 6 | 0.54 | -1.67, 2.75 |  |
| 6.Stigmatisation (0-12) |  |  |  | 0.58 |  |  |  | 0.33 |
| *CAT1* | 875 | 0.97 | 0.84,1.10 |  | 622 | 0.01 | -0.11, 0.14 |  |
| *CAT2* | 131 | 0.98 | 0.61, 1.35 |  | 98 | 0.08 | -0.30, 0.46 |  |
| *CAT3* | 67 | 1.29 | 0.72, 1.86 |  | 47 | 0.06 | -0.42, 0.54 |  |
| *CAT4* | 16 | 0.76 | 0.18, 1.34 |  | 9 | -0.09 | -1.02, 0.83 |  |
| *CAT5* | 16 | 1.61 | 0.49, 2.74 |  | 6 | -0.84 | -1.59, -0.09 |  |
| 7.Introvert (0-18) |  |  |  | 0.72 |  |  |  | 0.18 |
| *CAT1* | 874 | 2.56 | 2.37, 2.76 |  | 621 | -0.04 | -0.22, 0.14 |  |
| *CAT2* | 133 | 2.65 | 2.10, 3.21 |  | 98 | 0.05 | -0.49, 0.59 |  |
| *CAT3* | 68 | 2.59 | 1.91, 3.26 |  | 45 | 0.90 | 0.15, 1.64 |  |
| *CAT4* | 16 | 2.05 | 1.00, 3.10 |  | 9 | 0.37 | -0.64,1.39 |  |
| *CAT5* | 15 | 2.24 | 1.23, 3.24 |  | 6 | 0.53 | -0.89, 1.95 |  |
| 8.Harms of smoking (0-6) |  |  |  | 0.49 |  |  |  | 0.08 |
| *CAT1* | 885 | 1.97 | 1.85, 2.09 |  | 628 | 0.01 | -0.10, 0.12 |  |
| *CAT2* | 133 | 1.94 | 1.64, 2.25 |  | 97 | 0.17 | -0.12, 0.47 |  |
| *CAT3* | 68 | 2.12 | 1.65, 2.60 |  | 47 | 0.59 | 0.23, 0.95 |  |
| *CAT4* | 16 | 2.13 | 1.11, 3.16 |  | 9 | -0.04 | -0.85, 0.77 |  |
| *CAT5* | 16 | 2.19 | 1.43, 2.95 |  | 6 | 0.04 | -2.04, 2.11 |  |
| 9. Self-blame (0-15) |  |  |  | 0.55 |  |  |  | 0.28 |
| *CAT1* | 881 | 2.99 | 2.74, 3.24 |  | 620 | 0.16 | -0.07, 0.39 |  |
| *CAT2* | 132 | 3.59 | 2.80, 4.38 |  | 98 | 0.12 | -0.50, 0.73 |  |
| *CAT3* | 67 | 3.15 | 2.18, 4.11 |  | 46 | 1.07 | 0.30, 1.85 |  |
| *CAT4* | 16 | 2.54 | 1.02, 4.05 |  | 9 | 1.04 | -1.28, 3.37 |  |
| *CAT5* | 16 | 3.21 | 1.93, 4.50 |  | 6 | -0.05 | -2.09, 2.00 |  |
| 9.1. Self-blame (0-12)  *Modified scale* |  |  |  | 0.52 |  |  |  | 0.36 |
| *CAT1* | 881 | 2.35 | 2.15, 2.55 |  | 622 | 0.12 | -0.07, 0.30 |  |
| *CAT2* | 132 | 2.84 | 2.21, 3.46 |  | 98 | 0.09 | -0.40, 0.58 |  |
| *CAT3* | 67 | 2.45 | 1.68, 3.21 |  | 47 | 0.79 | 0.18, 1.41 |  |
| *CAT4* | 16 | 2.05 | 0.83, 3.26 |  | 9 | 0.64 | -1.17, 2.44 |  |
| *CAT5* | 16 | 2.69 | 1.68, 3.70 |  | 6 | 0.09 | -1.71, 1.89 |  |
| Kept busy to take my mind off things (0-3) |  |  |  | 0.84 |  |  |  | 0.65 |
| *CAT1* | 885 | 0.44 | 0.39, 0.49 |  | 622 | 0.06 | -0.01, 0.12 |  |
| *CAT2* | 133 | 0.42 | 0.29, 0.54 |  | 98 | 0.08 | -0.06, 0.23 |  |
| *CAT3* | 69 | 0.49 | 0.32, 0.67 |  | 47 | 0.14 | -0.16, 0.44 |  |
| *CAT4* | 16 | 0.49 | 0.10, 0.88 |  | 8 | 0.16 | -0.40, 0.72 |  |
| *CAT5* | 16 | 0.44 | 0.10, 0.79 |  | 6 | -0.09 | -0.25, 0.07 |  |
| Less interest in sex (0-3) |  |  |  | 0.44 |  |  |  | 0.32 |
| *CAT1* | 723 | 0.57 | 0.49, 0.64 |  | 514 | 0.03 | -0.05, 0.11 |  |
| *CAT2* | 109 | 0.63 | 0.41, 0.84 |  | 75 | 0.07 | -0.14, 0.27 |  |
| *CAT3* | 51 | 0.73 | 0.39, 1.08 |  | 35 | 0.41 | 0.06, 0.77 |  |
| *CAT4* | 12 | 0.42 | 0.07, 0.77 |  | 6 | 0.16 | -0.20, 0.53 |  |
| *CAT5* | 10 | 0.66 | 0.16, 1.17 |  | 4 | -0.03 | -0.49, 0.44 |  |

Table S2. Unadjusted mean scores from after screening results for COS LC Part 2

| Scales | N | Mean score | 95% CI | p-value (difference between CATs after screening) |
| --- | --- | --- | --- | --- |
| 10. Lung cancer (0-4) |  |  |  | 0.28 |
| *CAT1* | 617 | 1.34 | 1.25, 1.43 |  |
| *CAT2* | 20 | 0.75 | 0.32, 1.18 |  |
| *CAT3* | 14 | 2.05 | 1.16, 2.95 |  |
| *CAT4* | 5 | 1.27 | 0.26, 2.28 |  |
| *CAT5* | 3 | 1.48 | 0.60, 2.37 |  |
| 11. Relaxed/Calm (0-6) |  |  |  | 0.03 |
| *CAT1* | 618 | 1.26 | 1.16, 1.37 |  |
| *CAT2* | 20 | 0.60 | 0.12, 1.08 |  |
| *CAT3* | 15 | 2.45 | 1.64, 3.26 |  |
| *CAT4* | 6 | 1.95 | 1.32, 2.59 |  |
| *CAT5* | 5 | 0.96 | 0.13, 1.79 |  |
| 12. Social Relations (0- 6) |  |  |  | 0.17 |
| *CAT1* | 620 | 0.16 | 0.11, 0.21 |  |
| *CAT2* | 20 | 0.17 | -0.12, 0.45 |  |
| *CAT3* | 16 | 0.88 | 0.45, 1.29 |  |
| *CAT4* | 6 | 0.63 | -0.09, 1.35 |  |
| *CAT5* | 5 | 0.17 | -0.14, 0.47 |  |
| 13. Existential values (0- 12) |  |  |  | 0.05 |
| *CAT1* | 619 | 1.94 | 1.76, 2.13 |  |
| *CAT2* | 20 | 1.72 | 0.44, 3.00 |  |
| *CAT3* | 15 | 3.50 | 2.40, 4.61 |  |
| *CAT4* | 6 | 3.53 | 2.35, 4.72 |  |
| *CAT5* | 5 | 2.94 | 1.95, 3.93 |  |
| 14. Impulsivity (0- 12) |  |  |  | 0.29 |
| *CAT1* | 616 | 0.67 | 0.55, 0.79 |  |
| *CAT2* | 20 | 0.54 | -0.05, 1.14 |  |
| *CAT3* | 15 | 1.66 | 0.63, 2.70 |  |
| *CAT4* | 6 | 1.86 | 0.66, 3.07 |  |
| *CAT5* | 5 | 0.44 | -0.32, 1.20 |  |
| 15. Empathy (0- 6) |  |  |  | 0.06 |
| *CAT1* | 618 | 0.55 | 0.48, 0.63 |  |
| *CAT2* | 20 | 0.42 | 0.02, 0,81 |  |
| *CAT3* | 15 | 1.27 | 0.82, 1.73 |  |
| *CAT4* | 6 | 1.04 | 0.06, 2.03 |  |
| *CAT5* | 5 | 1.41 | 0.77, 2.04 |  |
| 15.1 Empathy (0- 4)  *Modified scale (-Q15)* |  |  |  | 0.40 |
| *CAT1* | 619 | 0.40 | 0.34, 0.46 |  |
| *CAT2* | 20 | 0.32 | -0.01, 0.66 |  |
| *CAT3* | 15 | 0.74 | 0.20, 1.28 |  |
| *CAT4* | 6 | 0.68 | 0.05, 1.32 |  |
| *CAT5* | 5 | 0.90 | 0.28, 1.52 |  |
| 15.2 Empathy (0- 4)  *Modified scale (-Q18)* |  |  |  | 0.13 |
| *CAT1* | 618 | 0.32 | 0.27, 0.37 |  |
| *CAT2* | 20 | 0.21 | -0.02, 0.44 |  |
| *CAT3* | 15 | 0.79 | 0.45, 1.12 |  |
| *CAT4* | 6 | 0.60 | 0.00, 1.19 |  |
| *CAT5* | 5 | 0.72 | 0.36, 1.09 |  |
| 16. Regretful still smoking, if current smoker (0-5) |  |  |  | 0.44 |
| *CAT1* | 235 | 4.31 | 3.66, 4.96 |  |
| *CAT2* | 5 | 2.94 | 1.22, 4.66 |  |
| *CAT3* | 8 | 4.62 | 3.35, 5.90 |  |
| *CAT4* | 1 | 6 | 6, 6 |  |
| *CAT5* | 1 | 2 | 2, 2 |  |
